# Multi-Marker Longitudinal Algorithms Incorporating HE4 and CA125 in Ovarian Cancer Screening of Postmenopausal Women

**DOI:** 10.3390/cancers12071931

**Published:** 2020-07-17

**Authors:** Aleksandra Gentry-Maharaj, Oleg Blyuss, Andy Ryan, Matthew Burnell, Chloe Karpinskyj, Richard Gunu, Jatinderpal K. Kalsi, Anne Dawnay, Ines P. Marino, Ranjit Manchanda, Karen Lu, Wei-Lei Yang, John F. Timms, Mahesh Parmar, Steven J. Skates, Robert C. Bast, Ian J. Jacobs, Alexey Zaikin, Usha Menon

**Affiliations:** 1 MRC Clinical Trials Unit at UCL, Institute of Clinical Trials & Methodology, London WC1V 6LJ, UK; a.gentry-maharaj@ucl.ac.uk (A.G.-M.); a.ryan@ucl.ac.uk (A.R.); m.burnell@ucl.ac.uk (M.B.); c.karpinskyj@ucl.ac.uk (C.K.); r.manchanda@qmul.ac.uk (R.M.); m.parmar@ucl.ac.uk (M.P.); 2 School of Physics, Astronomy and Mathematics, University of Hertfordshire, Hatfield AL10 9AB, UK; o.blyuss@qmul.ac.uk; 3 Department of Paediatrics and Paediatric Infectious Diseases, Sechenov First Moscow State Medical University, Moscow 119991, Russia; alexey.zaikin@ucl.ac.uk; 4 Department of Women’s Cancer, Institute for Women’s Health, University College London, London WC1E 6BT, UK; r.gunu@ucl.ac.uk (R.G.); j.k.kalsi@ucl.ac.uk (J.K.K.); john.timms@ucl.ac.uk (J.F.T.); i.jacobs@unsw.edu.au (I.J.J.); 5 Clinical Biochemistry, Barts Health NHS Trust, London E1 8PR, UK; anne.dawnay@bartshealth.nhs.uk; 6 Department of Biology and Geology, Physics and Inorganic Chemistry, Universidad Rey Juan Carlos, 28933 Madrid, Spain; ines.perez@urjc.es; 7 Wolfson Institute of Preventive Medicine, Barts CRUK Cancer Centre, Queen Mary University of London, London EC1M 6BQ, UK; 8 Department of Gynaecological Oncology, St Bartholomew’s Hospital, Barts Health NHS Trust, London EC1A 7BE, UK; 9 University of Texas M.D. Anderson Cancer Center, Houston, TX 77030, USA; khlu@mdanderson.org (K.L.); wlyang@mdanderson.org (W.-L.Y.); rbast@mdanderson.org (R.C.B.J.); 10 Massachusetts General Hospital and Harvard Medical School, Boston, MA 02114, USA; sskates@mgh.harvard.edu; 11 University of New South Wales, Sydney 2052, Australia; 12 Department of Applied Mathematics, Lobachevsky University of Nyzhniy Novgorod, Nizhniy Novgorod 603105, Russia; 13 Department of Mathematics, University College London, London WC1H 0AY, UK

**Keywords:** ovarian cancer, CA125, HE4, UKCTOCS, MMT, screening, postmenopausal women

## Abstract

Longitudinal CA125 algorithms are the current basis of ovarian cancer screening. We report on longitudinal algorithms incorporating multiple markers. In the multimodal arm of United Kingdom Collaborative Trial of Ovarian Cancer Screening (UKCTOCS), 50,640 postmenopausal women underwent annual screening using a serum CA125 longitudinal algorithm. Women (cases) with invasive tubo-ovarian cancer (WHO 2014) following outcome review with stored annual serum samples donated in the 5 years preceding diagnosis were matched 1:1 to controls (no invasive tubo-ovarian cancer) in terms of the number of annual samples and age at randomisation. Blinded samples were assayed for serum human epididymis protein 4 (HE4), CA72-4 and anti-TP53 autoantibodies. Multimarker method of mean trends (MMT) longitudinal algorithms were developed using the assay results and trial CA125 values on the training set and evaluated in the blinded validation set. The study set comprised of 1363 (2–5 per woman) serial samples from 179 cases and 181 controls. In the validation set, area under the curve (AUC) and sensitivity of longitudinal CA125-MMT algorithm were 0.911 (0.871–0.952) and 90.5% (82.5–98.6%). None of the longitudinal multi-marker algorithms (CA125-HE4, CA125-HE4-CA72-4, CA125-HE4-CA72-4-anti-TP53) performed better or improved on lead-time. Our population study suggests that longitudinal HE4, CA72-4, anti-TP53 autoantibodies adds little value to longitudinal serum CA125 as a first-line test in ovarian cancer screening of postmenopausal women.

## 1. Introduction

Ovarian cancer is the most fatal of all gynaecological malignancies [1]. Despite significant advances in treatment, the impact on mortality over the past three decades has been modest [2,3,4,5]. A key contributing factor is diagnosis at advanced stages when survival is poor (5-year survival rates for stage III/IV disease 35% versus 90% for stage I) [6]. Efforts over the past four decades have therefore focused on early detection. Advances in understanding the natural history has meanwhile clarified the need to focus on detecting invasive tubo-ovarian cancer (WHO 2014), especially Type II (high-grade serous) cancers as they account for most of the mortality.

Since its discovery in 1981, CA125 remains the best performing marker for ovarian cancer. It forms an integral part of differential diagnosis and has been studied extensively in the context of screening [7,8,9,10]. In screening, performance has been improved by the use of the Risk of Ovarian Cancer Algorithm (ROCA), which assesses serial changes in CA125 over time. As a first-line test for ovarian cancer screening, ROCA had a sensitivity of 85.8% in the United Kingdom Collaborative Trial of Ovarian Cancer Screening (UKCTOCS) during incidence screening [11]. Moreover, screening using the multimodal strategy (ROCA as the first-line test with transvaginal ultrasound as the second-line test) resulted for the first time in a stage shift in women diagnosed with invasive tubo-ovarian cancer compared with no screening, on an “intention to screen” analysis. The mortality benefit was however not definitive at the first analysis and further follow-up is underway [5]. Retrospective analysis using data from the trial suggests that other longitudinal CA125 algorithms such as parametric empirical Bayes (PEB) [12,13], parenclitic networks [14], deep learning [15] and method of mean trends (MMT) [16,17] are likely to perform similarly.

Over the years, data from small case–control studies [18,19] have suggested that markers like human epididymis protein 4 (HE4) and TP53 autoantibodies might complement CA125 in ovarian cancer screening. HE4 was the second best marker for invasive tubo-ovarian cancer after CA125 (sensitivity CA125 86%; HE4 73%) in a nested case–control study within the Prostate, Lung, Colorectal and Ovarian cancer screening (PLCO) trial using a single preclinical sample taken within 6 months of diagnosis [20]. Data from the Carotene and Retinol Efficacy Trial suggested that a panel including CA125, HE4 and mesothelin may provide a signal for ovarian cancer 3 years before diagnosis [21]. More recently, elevated anti-TP53 autoantibody levels were detected in 16% of cases not detected by ROCA in the UKCTOCS sample set, providing in these cases a lead time of 22 months [19]. Other studies have explored the performance of multi-marker (CA125, IGFBP2, LCAT, SHBG, GRP78 and calprotectin) [22] panels. All have used cut-offs for interpreting results. Longitudinal algorithms incorporating multiple markers have not been previously investigated.

We report on the performance of longitudinal multi-marker algorithms incorporating CA125, HE4, CA72-4 and anti-TP53 autoantibodies as a first-line test in ovarian cancer screening using the prospective specimen collection and the retrospective blinded evaluation (PRoBe) design [23] within the general population UKCTOCS trial.

## 2. Results

During a median follow-up of 11.1 (IQR 10.0–12.0) years, of the 46,237 women randomised to the multimodal screening (MMS) arm of UKCTOCS who had two or more annual screens, 238 were diagnosed with invasive tubo-ovarian cancer [24]. At the time of sample selection, 179 (75.2%) of the latter had adequate (>2 mL) serum in the biorepository. The final set comprised of 179 cases and 181 controls. Training and validation sets comprised of 181 women (90 invasive tubo-ovarian cancer cases, 91 controls; 676 annual samples) and 179 women (89 invasive tubo-ovarian cancer cases, 90 controls; 677 annual samples), respectively (Table 1), with 2–5 serial samples per woman.

Of the cases, 68 and 74 were diagnosed within 1 year of sample in the training and validation set, respectively (Table 1). There was no difference in age between the cases and controls. Baseline and clinical characteristics of the women in the training and validation sets were well balanced (Table 2). There were 13 Type I, 74 Type II, and 3 Type uncertain tubo-ovarian cancers in the training set, and 11 Type I, 72 Type II, and 6 Type uncertain in the validation set. In the training set, four longitudinal multi-marker algorithms (CA125-HE4-MMT1, CA125-HE4-MMT2, CA125-HE4-CA72-4-MMT, CA125-HE4-CA72-4-anti-TP53-MMT) were derived and then applied to the validation set, which comprised of 670 annual samples from 179 women (Table 1).

**Figure 1:**
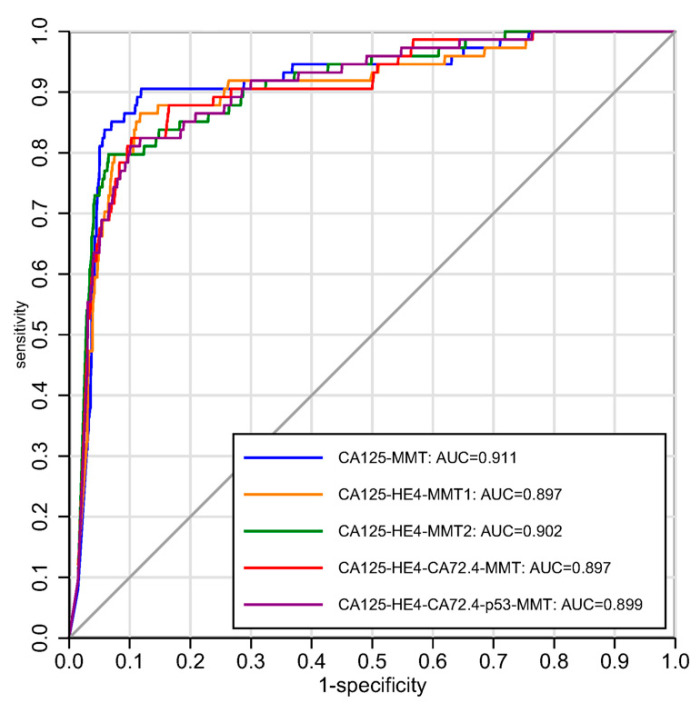
ROC curves with the AUC for each of the longitudinal algorithms.

For the detection of invasive tubo-ovarian cancers diagnosed within 1 year of last annual sample, at a fixed specificity of 87.6% (similar to ROCA as a first-line test in UKCTOCS), CA125, HE4 or CA72-4 alone had a sensitivity of 73%, 58.1%, and 37.8%, respectively (Table 3). Figure 1 shows the Receiver Operating Curve (ROC) for the performance of CA125-MMT versus the four newly developed models, CA125-HE4-MMT1, CA125-HE4-MMT2, CA125-HE4-CA72-4-MMT, CA125-HE4-CA72-4-anti-TP53-MMT, in the validation set. CA125-MMT provided a higher area under the curve (AUC) compared with any other model (0.911 versus 0.897–0.902) (Table 3). At a specificity of 87.6%, CA125-MMT outperformed all other multimarker models (sensitivity of 90.5% versus 81–86.5%) with CA125-HE4-MMT1 being the next best model.

Of the 74 invasive tubo-ovarian cancers in the validation set, 67 (90.5%) were detected by the CA125-MMT model, of whom 53 (79.1%) were Type II cancers (Table S1). Of the other models, the CA125-HE4-MMT1 detected 64 cancers with one additional woman with Type I cancer not detected by the CA125-MMT model.

In the lead time analysis, no multimarker algorithm outperformed CA125-MMT. The lead time from marker elevation/change point to diagnosis was on average 140–148 days (multimarker algorithms) compared with 152 days (CA125-MMT algorithm) (Table 4).

The newer models offered no benefit in detecting poor prognostic cases (who died within 5 years of the last sample taken), with 12 of 67 (18.0%) women detected by the CA125-MMT model. The CA125-HE4-MMT2 model was able to detect 11 cases who died but at a cost of only detecting 60 cases. None of the other models were able to improve on this.

## 3. Discussion

### 3.1. Principal Findings

This is the first study to explore the added value of longitudinal multi-marker profiles to longitudinal CA125 for ovarian cancer screening. In this population-based case–control study, the addition of longitudinal HE4 or other markers such as CA72-4 and anti-TP53 did not improve on the performance of the longitudinal single marker CA125 algorithm in postmenopausal women [16]. There was also no improvement in lead time over longitudinal CA125. It would therefore be hard to justify the higher costs of including HE4 alongside CA125 in this population.

### 3.2. Results in Context

The current sensitivity of multimodal ovarian cancer screening is 87%. The MMS strategy consists of first-line screening using the longitudinal CA125 algorithm (ROCA) followed by repeat CA125 profiling and transvaginal ultrasound in women with intermediate or elevated test results. There has been longstanding interest in the possibility of increasing sensitivity by adding other markers that might help detect the 15% of the cases that are currently missed using the MMS strategy, which is probably due to the tumours not expressing CA125. HE4 has been the fore runner among potential markers ever since being highlighted in the study by Cramer and colleagues [20] using samples from the ovarian cancer screening arm of the PLCO trial. This study showed that out of 35 markers evaluated in a single sample taken 6 months prior to diagnosis in 118 women with ovarian cancer, HE4 (sensitivity 73%) was the second best marker to CA125 (sensitivity 86%). A previous exploratory study nested within the UKCTOCS cohort which was enriched for the missed cases also seemed to suggest that longitudinal HE4 and CA72-4 might improve sensitivity [25]. In the same UKCTOCS nested case–control study, p53 autoantibody profile was shown to complement CA125 in that it was able to detect 20.7% of those not detected by ROCA [19]. This was however not borne out in our rigorous study that used all available samples from the multimodal cohort. Our results using a cut-off as used in the PLCO analysis resulted in similar results (HE4 sensitivity 58.1% vs. 73% in PLCO; CA125 73% versus 86% in PLCO).

### 3.3. Clinical and Research Implications

There are now a number of longitudinal CA125 algorithms [16,26]. The advantage of the MMT methodologies presented here is that they incorporate longitudinal profiling of multiple biomarkers in a single algorithm. This sets the stage for future work incorporating novel markers as they gain recognition in ovarian cancer screening. Moreover, the longitudinal algorithms framework described here is applicable to other cancers and diseases where a serial profile of multiple markers is available.

### 3.4. Strengths and Limitations

The major strength is the decrease in bias through the use of a population-based nested case–control as per the PRoBE study design [23]. All samples were prospectively collected before outcome ascertainment. Linkage to electronic health records and independent outcome review of cases ensured complete and accurate data. The study has involved the largest dataset of serial samples up to 5 years prior to diagnosis of tubo-ovarian cancer in postmenopausal women from the general population that we are aware of. While the number of cases may seem small, the set consisted of 75% of all women who had two or more annual screens and were diagnosed with invasive tubo-ovarian cancer in the course of 343,156 screens. CA125, HE4 and CA72-4 assays were assayed by ELISA (enzyme-linked immunosorbent assay), the gold standard [27] in assaying markers. When applying the algorithm in the validation set, the statistician was blinded to the outcome. The algorithms described have a flexible modelling framework and hence can be used more widely. HE4 levels are known to increase with age in healthy people [28]. To address this in our design we have used age-matched controls. Moreover, as our models are based on trend indices rather than raw HE4 levels, the variation in age is unlikely to affect our results. While all women donated annual samples, it needs to be noted that the available serial repeat samples were influenced by the use of CA125 and ROCA in the trial. The results cannot be extrapolated to high-risk screening strategies where the frequency of screening is 6-monthly or less. In these high risk populations, ovarian cancer screening, if undertaken, usually starts at age 35 and in these premenopausal women HE4 may be helpful in ruling out endometriosis. We only used one control per case due to limited funds. Including a larger number of controls would have shed more light on the biological variation of HE4 in postmenopausal women.

## 4. Materials and Methods

### 4.1. Subjects

Between April 2001 and September 2005, 202,638 postmenopausal women aged 50–74 were recruited to UKCTOCS through 13 trial centres based in England, Wales and Northern Ireland (NI). The women were randomised to annual screening: (1) MMS using serum CA125 interpreted with the ROCA followed by transvaginal ultrasound (TVS) as a second-line test (*n* = 50,640); (2) ultrasound (USS) screening using TVS alone (*n* = 50,639); or (3) no screening (control) group (*n* = 101,359) in a 1:1:2 ratio, as described previously [29,30]. In the MMS group, based on ROCA, women were triaged to (1) annual screening if normal; (2) repeat CA125 in 6 weeks if intermediate; (3) repeat CA125 and TVS if the risk was elevated. Women with abnormal ultrasound or persistent elevated risk (irrespective of scan findings) had clinical assessment by a trial clinician and additional investigations within the NHS. Women who had surgery or biopsy for suspected tubo-ovarian cancer after clinical assessment were considered trial-screen positive. Blood samples were taken at the trial centres in gel tubes (8 mL gel separation serum tubes; Greiner Bio-One 455071, Stonehouse, UK) and transported at room temperature overnight to the central UKCTOCS laboratory using the standard protocol [11,29,31]. The samples received within 56 h of venepuncture were processed by centrifuging at 1500× *g* for 10 min. The serum was separated and assayed for CA125. The excess serum was pre-cooled at −80 °C and stored in 500 µL straws in liquid nitrogen at an off-site cryorepository until the sample was retrieved and thawed for the current analysis.

UKCTOCS was approved by the UK North West Multicentre Research Ethics Committees (North West MREC 00/8/34) on 21 June 2000 with site-specific approval from the local regional ethics committees and the Caldicott guardians (data controllers) of the primary care trusts. The current study was approved by the NRES (National Research Ethics Service) Committee North East-Tyne & Wear South (Ref: 15/NE/0025) on 20 January 2015.

All women were followed up through linkage via electronic health records for cancers and deaths as previously detailed [5,30] (NHS Digital, England and Wales; Northern Ireland Cancer Registry and NI Health and Social Care Business Services Organisation, NI). Women also completed two follow-up postal questionnaires; 3–5 years after randomisation, and in April 2014. As for the mortality analysis previously undertaken [5], cancer registrations received up to 5 April 2015 (England, Wales), and 9 April 2015 (NI) were used.

For all women with a possible diagnosis of ovarian cancer (one of 19 ICD-10 codes) [5], medical notes were requested and reviewed by a member of the independent Outcomes Review Committee (two pathologists and two gynaecological oncologists) who were masked to the randomisation group [5]. The Outcomes Review Committee [5] confirmed the final diagnosis—the primary cancer site (WHO 2014) [24]; the stage and morphology; and where possible, classified invasive tubo-ovarian cancer (WHO 2014 classification) [24] which included epithelial ovarian, fallopian tube and the primary peritoneal cancer as per WHO 2003 classification into Type I (low-grade serous, low-grade endometrioid, mucinous, clear cell) or Type II (high-grade serous, high-grade endometrioid, carcinosarcomas, undifferentiated) cancers or Type uncertain [32].

### 4.2. Sample Set and CA125, HE4, CA72-4 and Anti-TP53 Autoantibody Assays

The cases were all women in the MMS group diagnosed with invasive tubo-ovarian cancer [24] during follow-up, who had ≥2 serial samples taken within 5 years of diagnosis. Women with borderline epithelial and non-epithelial ovarian cancer were excluded. The controls were randomly chosen from the remaining women who did not have primary malignant neoplasm of the ovary. The cases were matched (1:1) to controls in terms of the number of annual samples available and age (±6 months) at randomisation.

The sample set for the study included all serial samples in the cases and controls where >2 mL serum was available in the cryorepository.

Once assayed for CA125 (Roche Diagnostics, Burgess Hill, UK), the excess serum was stored in liquid nitrogen in an off-site commercial cryorepository until it was retrieved for this study.

CA125 measurements (Roche, Burgess Hill, UK) completed as part of the UKCTOCS screening protocol were used along with anti-TP53 autoantibody values assayed during a previously reported study [19]. HE4 and CA72-4 (Roche Diagnostics, Burgess Hill) were assayed on all samples using the Roche Cobas analyser at the UCL Department of Women’s Cancer Proteomics laboratory.

### 4.3. Method of Mean Trends (MMT) Algorithms Incorporating CA125, HE4, CA72-4 and Anti-TP53 Autoantibody

The MMT that evaluates the dynamics of longitudinal CA125 measurements has been described previously [16].

In brief, the serial pattern of a particular biomarker $Y_{i,j},\;j=1\ldots T$ of each woman “i” was mapped into a five-variable space. The new variables included the mean derivative weighted to the most recent measurement, the mean area under the time series (1), the coefficient of variation (2), the “centre of mass” of the time series (3) and the most recent measurement.

(1) $$\left(\sum_{j=1}^{T-1}\frac{\left(Y_{i,j+1}-Y_{i,j}\right)\left(t_{i,j+1}-t_{i,j}\right)}{2}\right)/\left(T-1\right)$$

(2) $$\sqrt{\frac{\sum_{j=1}^{T}{\left(Y_{i,j}-\bar{Y_{i}}\right)}^{2}}{T}}/\bar{Y_{i}}$$

(3) $$\frac{\sum_{j=1}^{T}Y_{i,j}t_{i,j}}{\sum_{j=1}^{T}t_{i,j}}$$

The mean derivative was evaluated as $\sum_{j=1}^{T-1}w_{ij}\frac{Y_{i,j+1}-Y_{i,j}}{t_{i,j+1}-t_{i,j}}$ where weights $w_{j}$ were computed for each interval between two consecutive measurements as $w_{ij}=\frac{1}{t_{i,T}-\left(t_{i,j+1}+t_{i,j}\right)/2}$. Here, $t_{i,T}$ was the age of the patient at the time of the most recent sample, while $t_{i,j}$ was age of the patient when the j-th sample was taken.

To use this approach to incorporate multiple serial biomarkers, for each of the proteins, the aforementioned five variables were evaluated and combined together into a logistic regression with AIC (Akaike information criterion) used to select the predictors that explain the labels of the patients (control = 0, case = 1) in the most optimal way.

With the MMT approach, we generated four separate models for the prediction of the risk of ovarian cancer using the serial measurements of multiple biomarkers:

- CA125-HE4-MMT1, where variable selection was made only over HE4 indices added to the reported CA125-MMT model [16];
- CA125-HE4-MMT2, where five indices for both CA125 and HE4 were used with further variable selection;
- CA125-HE4-CA72-4-MMT, where five indices for CA125, HE4 and CA72-4 were used with further variable selection;
- CA125-HE4-CA72-4- anti-TP53-MMT, where five indices for CA125, HE4, CA72-4 and anti-TP53 were used with further variable selection.

The performance of these models was evaluated against the original CA125-MMT approach as well as the actual biomarkers levels.

### 4.4. Statistical Analysis

The cases and controls were randomly split into “training” and “validation” sets in a 1:1 ratio. Longitudinal CA125 MMT described previously [16], and four separate longitudinal multi-marker (CA125-HE4-MMT1, CA125-HE4-MMT2, CA125-HE4-CA72-4-MMT and CA125-HE4-CA72-4-anti-TP53-MMT) algorithms were built using all available serial samples from the cases and controls in the “training” set by OB. OB then applied them to the blinded “validation” set.

Statistical analysis was undertaken by MB to ensure blinding. The performance characteristics of the newly constructed algorithms as a first-line test were evaluated and compared with the CA125-MMT in terms of the following: (1) sensitivity at a fixed specificity of 87.6% similar to ROCA in UKCTOCS [11]; (2) average lead time for all cases detected (the date of detection is the date when risk given by algorithm is abnormal and for all further annual measurements it remains abnormal); (3) the area under the receiver operating characteristic (ROC) curve (AUC). Inference for the ROC curves was based on cluster-robust standard errors that accounted for the serially correlated nature of the samples [33]. At fixed specificity (87.6%), the performance characteristics of CA125 and HE4 cut-offs were compared with those of the newly derived algorithm.

Only annual samples were included in this analysis. The last blood sample was considered as true positive (if within 1 year from diagnosis) and all prior annual samples as true negatives. In the controls, all samples were included as true negatives.

To investigate whether any of the algorithms identified invasive tubo-ovarian cancer cases earlier than CA125-MMT, we performed lead time analysis, where the mean interval from detection to diagnosis was compared for all algorithms. For each algorithm, the average interval was calculated in days for only those cases that were detected as abnormal by the algorithm. Here, we assumed that an algorithm identified a cancer case at a particular measurement if both at this and at all subsequent measurements it classified the risk as abnormal. A further analysis explored how many of the cancers missed by ROCA during the trial (trial-screen negative cases) would have been detected by each of the algorithms.

## 5. Conclusions

In the context of screening, our study suggests that the additional value of HE4, CA72-4 and p53 autoantibodies to CA125 as a first line test in screening for ovarian cancer of postmenopausal women from the general population is limited. Further work on the value of these markers as a reflex test following elevated risk may show promise and strengthen the confidence in cancer diagnosis and thus shorten the period between the screening test and surgical intervention.

## Figures and Tables

**Table 1:** Details of cases (invasive tubo-ovarian cancer) and controls in training and validation sets.

Group	Overall		Annual Samples Available in Year Preceding Diagnosis	
	No. of Women	No. of Annual Samples	No. of Women	No. of Annual Samples
**Training Set**				
Cases	90	317	68	68
Controls	91	359	113/167 *	608
**Validation Set**				
Cases	89	332	74	74
Controls	90	355	105/173 *	613

* a case is included as a control until the screen is within a year of diagnosis for the purposes of this analysis—the first number is unique controls and the second number includes those who will become cases.

**Table 2:** Characteristics of cases and controls in training and validation sets.

Baseline Characteristics	Training Set	Validation Set
No. of women	181	179
Median age at recruitment (years)	63.54	63.68
BMI	26.46	25.99
OCP use	90 (49.7%)	88 (49.2%)
Median Duration of OCP use (years)	5 (*n* = 89)	5 (*n* = 86)
Hysterectomy	35 (19.3%)	34 (19.0%)
% White ethnicity	177 (97.8%)	174 (97.6%)
HRT use	25 (13.8%)	33 (18.4%)
Personal history of breast cancer	3 (1.66%)	7 (3.91%)
**Morphology of Cases**		
Invasive tubo-ovarian cancer	90	89
**Histological Type of Invasive Tubo-Ovarian Cancer**		
**Type I**	***13***	***11***
Endometrioid (low grade)	6	5
Serous (low grade)	1	2
Clear cell	6	4
**Type II**	***68***	***63***
High grade serous ovarian	57	62
Carcinoma, NOS	10	3
Endometrioid (high grade)	6	5
Carcinosarcoma	1	2
**Type uncertain**	***3***	***6***
Carcinoma, NOS	2	4
Serous (grade unknown)	1	2
**Stage of Invasive Tubo-Ovarian Cancer**		
I	21	20
II	12	10
III	47	53
IV	10	6

BMI, body mass index; OCP, oral contraceptive pill; HRT, hormone replacement therapy.

**Table 3:** Sensitivity and area under the ROC curve (AUC) of algorithms for the detection of invasive tubo-ovarian cancer diagnosed within one year of sample in the validation set.

Algorithms	AUC (95%CI)	Sensitivity (95%CI) at 87.6% SPECIFICITY
**CA125-MMT**	91.1	90.5
	(87.1 to 95.2)	(82.5 to 98.6)
**CA125-HE4-MMT1**	89.7	86.5
	(85.6 to 93.8)	(77.7 to 95.2)
**CA125-HE4-MMT2**	90.2	81
	(86.4 to 94)	(71.8 to 90.4)
**CA125-HE4-CA72-4-MMT**	89.7	82.4
	(85.8 to 93.7)	(73.5 to 91.4)
**CA125-HE4-CA72-4-anti-TP53-MMT**	90	82.4
	(86.2 to 93.6)	(73.5 to 91.4)
**CA125**	86.5	73
	(81.1 to 91.9)	(61.1 to 84.8)
**HE4**	80.4	58.1
	(74.8 to 86)	(45.4 to 70.8)
**CA72-4**	71.7	37.8
	(65 to 78.5)	(22.9 to 49.8)

AUC, area under ROC curve; CI, confidence interval.

**Table 4:** Lead time of algorithms for the detection of invasive tubo-ovarian cancer in the validation set.

Algorithm	No. of Cases Detected by Algorithm	Mean Lead Time	SD
**CA125-MMT**	67	152	95
**CA125-HE4-MMT1**	64	148	95
**CA125-HE4-MMT2**	60	140	91
**CA125-HE4-CA72-4-MMT**	61	144	92
**CA125-HE4-CA72-4-anti-TP53-MMT**	61	144	92

## Supplementary Materials

The following are available online at https://www.mdpi.com/2072-6694/12/7/1931/s1, Table S1: Details of invasive epithelial cancers diagnosed within 1 year of sample detected/missed by algorithms in the validation set
