# Supplementary material for: Multi-Marker Longitudinal Algorithms Incorporating HE4 and CA125 in Ovarian Cancer Screening of Postmenopausal Women
Source: Cancers (Basel). 2020 Jul 17;12(7):1931. doi: 10.3390/cancers12071931 (PMC7409061; doi:10.3390/cancers12071931)
Supplement: Supplementary file 1 [file cancers-12-01931-s001.pdf]

Supplementary materials: Multi-Marker Longitudinal Algorithms Incorporating HE4 and CA125 in Ovarian Cancer Screening of Postmenopausal Women

Aleksandra Gentry-Maharaj, Oleg Blyuss, Andy Ryan, Matthew Burnell, Chloe Karpinskyj, Richard Gunu, Jatinderpal K. Kalsi, Anne Dawnay, Ines P. Marino, Ranjit Manchanda, Karen Lu, Wei-Lei Yang, John F. Timms, Max Parmar, Steven J. Skates, Robert C. Bast Jr, Ian J. Jacobs, Alexey Zaikin and Usha Menon

Table 1. Details of invasive epithelial cancers diagnosed within 1 year of sample detected/missed by algorithms in the validation set.

Algorithm	Detected by Algorithm				Missed by Algorithm			
	No. Detected	Type I	Type II	Type Uncertain	No. Missed	Type I	Type II	Type Uncertain
**CA125-MMT**	67	8	53	6	7	1	2	4
**CA125-HE4-MMT1**	64	9	49	6	10	0	6	4
**CA125-HE4-MMT2**	60	7	48	5	14	2	7	5
**CA125-HE4-CA72.4-MMT**	61	8	47	6	13	1	8	4
**CA125-HE4-CA72.4-anti-TP53-MMT**	61	8	47	6	13	1	8	4
**CA125 (35 U/mL)***	37	5	29	3	37	4	26	7
**CA125 (30 U/mL)***	42	5	34	3	32	4	21	7
**CA125 (22 U/mL)***	54	7	43	4	20	2	12	6
**HE4 (128 pml)***	29	3	23	3	45	6	32	7

* at 87.6% specificity

© 2020 by the authors. Licensee MDPI, Basel, Switzerland. This article is an open access article distributed under the terms and conditions of the Creative Commons Attribution (CC BY) license (http://creativecommons.org/licenses/by/4.0/).
